# Data on the effect of pro-fibrotic cytokine TGF-β on hepatic stellate cell autophagy

**DOI:** 10.1016/j.dib.2016.12.005

**Published:** 2016-12-08

**Authors:** Paul G. Thomes, Elizabeth Brandon-Warner, Ting Li, Terrence M. Donohue, Laura W. Schrum

**Affiliations:** a Department of Internal Medicine, Carolinas Medical Center, Charlotte, NC, USA; b Department of Internal Medicine, University of Nebraska Medical Center, Omaha, NE, USA

## Abstract

Our data describe autophagic flux in primary rat hepatic stellate cells (rHSCs) treated with pro-fibrotic growth factor, transforming growth factor beta (TGF-β). An autophagy flux experiment determines the rate of synthesis and degradation of the autophagosome marker, LC3-II in the presence and absence of the lysosomal inhibitor bafilomcyin, which blocks LC3-II degradation in lysosomes. The effects of a test agent on LC3-II flux through the autophagic pathway is determined immunochemically by its relative amounts detected in lysates of cells treated with and without bafilomycin. This measurement helps to validate whether exposure to an agent affects the biogenesis or the degradation of autophagosomes during autophagy, a major macromolecular degrading mechanism in eukaryotic cells. (“Rev-erb Agonist and TGF-β Similarly Affect Autophagy but Differentially Regulate Hepatic Stellate Cell Fibrogenic Phenotype” (Thomes et al., in press) [1].

**Specifications Table**

**Table:** 

Subject area	*Biology*
More specific subject area	*Biochemistry*
Type of data	*figure*
How data was acquired	*Western blotting*
Data format	*Analyzed*
Experimental factors	*Autophagy flux experiment was performed according to Klionsky et al.,*[2]
Experimental features	*Expression of autophagosome marker, LC3-II was determined in lysates of primary rat hepatic stellate cells treated with pro-fibrotic cytokine TGF-β in the presence and absence of lysosomal inhibitor, bafilomycin.*
Data source location	*Charlotte, NC, USA*
Data accessibility	*Data is with this article*

**Value of the data**

1. The data show LC3-II flux in primary rHSCs after treatment with the pro-fibrotic cytokine TGF-β in the presence or absence of bafilomycin.
2. Compared with untreated control and bafilomycin-only- treated cells respectively, TGF-β exposure decreases LC3-II protein in both the absence and in the presence of bafilomycin.
3. Future studies on the dynamics of TGF-β regulation of autophagy are essential to establish the relationship between autophagy and HSC fibrogenic phenotype, and the functional role of autophagy in liver fibrosis.

## 1 Data

The data reveal the content of LC3-II, an autophagosome marker protein in primary rHSC treated with or without TGF-β for 48 h in the presence and absence of bafilomycin A1 (treated during the final 4 h before harvest). Bafilomycin A1 inhibits the lysosome proton pump to prevent lysosome acidification, thereby blocking degradation of cargo, including LC3-II. This experiment is also known as LC3-II or autophagy flux experiment [2].

## 2 Experimental design, materials and methods

### 2.1 Materials

LC3-II antibody was from Cell Signaling Technology Inc. (Danvers MA). TGF-β was from R&D systems (Minneapolis, MN). We purchased bafilomycin A1 from Sigma (St. Louis MO).

### 2.2 Primary cell isolation

**Fig. 1:**
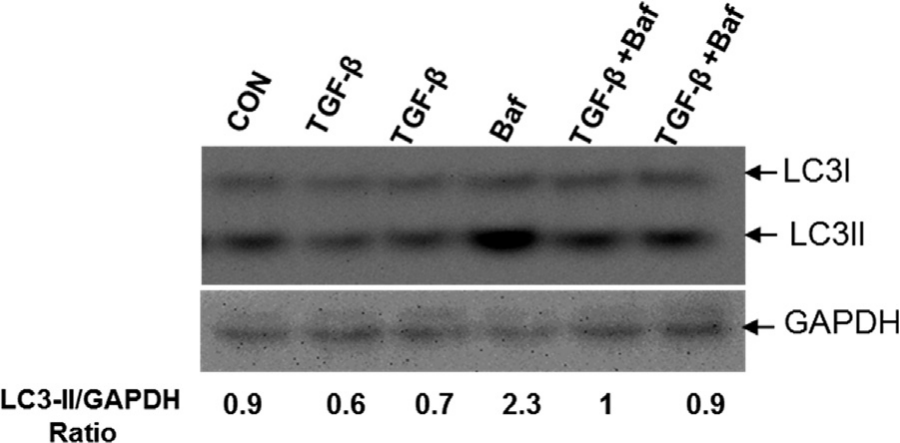
LC3-II flux in primary rat HSCs. Flux measurement of LC3-II in primary HSC treated with or without 5 ng TGF-β/ml for 48 h in the presence and absence of 100 nM bafilomycin A1 (treated during last 4 h before harvest). Similar results were obtained from 3 sets of independent experiments (*n*=2×3).

The animal studies subcommittee (IACUC) of the Carolinas Medical Center approved all animal protocols described here. We prepared primary rat hepatic stellate cells (HSC) by perfusing rat livers *in situ* with collagenase as described [3]. Following perfusion, HSCs from these animals were isolated by density gradient centrifugation (Fig. 1).

### 2.3 LC3-II flux

To assess the effects of the TGF-β on HCS autophagy, we measured LC3-II levels by western blot in primary rHSCs treated with or without 5 ng TGF-β/ml for 48 h in the presence or absence of 100 nM bafilomycin A1 (treated during last 4 h before harvest). Cells were treated in 0.2% serum containing medium. All cells were incubated at 37 °C in 4% CO_2_ atmosphere.
