# Supplementary material for: Data on the effect of pro-fibrotic cytokine TGF-β on hepatic stellate cell autophagy
Source: Data Brief. 2016 Dec 8;10:312–4. doi: 10.1016/j.dib.2016.12.005 (PMC5156597; doi:10.1016/j.dib.2016.12.005)
Supplement: Supplementary file 1 — Supplementary material [file mmc1.docx]

**AUTHOR DECLARATION TEMPLATE**

We wish to confirm that there are no known conflicts of interest associated with this publication and there has been no significant financial support for this work that could have influenced its outcome.

We confirm that the manuscript has been read and approved by all named authors and that there are no other persons who satisfied the criteria for authorship but are not listed. We further confirm that the order of authors listed in the manuscript has been approved by all of us.

We confirm that we have given due consideration to the protection of intellectual property associated with this work and that there are no impediments to publication, including the timing of publication, with respect to intellectual property. In so doing we confirm that we have followed the regulations of our institutions concerning intellectual property.

We further confirm that any aspect of the work covered in this manuscript that has involved experimental animals has been conducted with the ethical approval of all relevant bodies and that such approvals are acknowledged within the manuscript.

Sincerely,

**
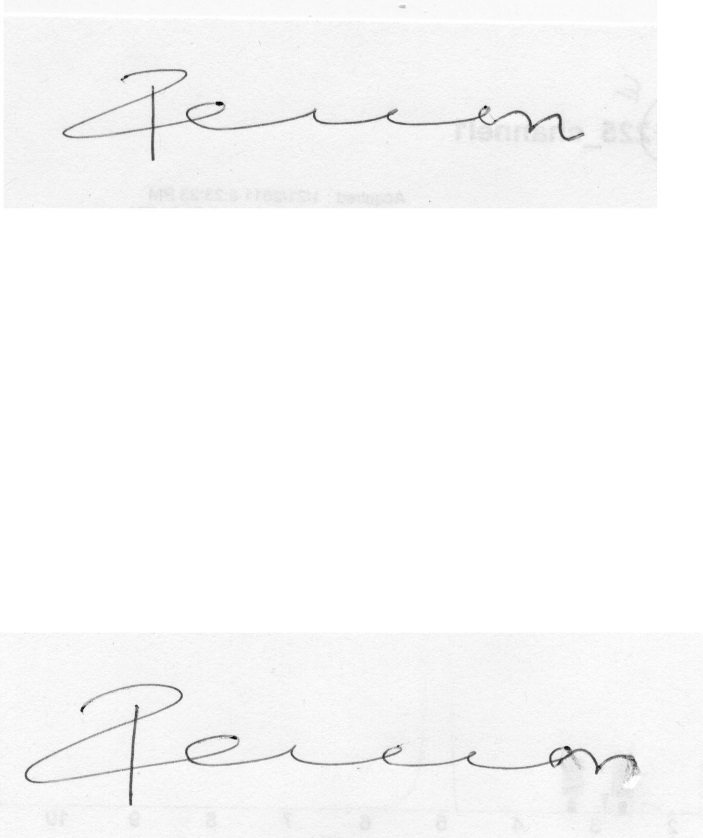
**

Paul Thomes, PhD

Research Scientist,

Dept of Internal Medicine, Carolinas Medical Center

Charlotte, NC, USA

Phone. 704-582-9491

Corresponding author signing on behalf of all other authors.
